# Supplementary material for: Feeding a rich diet supplemented with the translation inhibitor cycloheximide decreases lifespan and ovary size in Drosophila
Source: bioRxiv. 2024 Aug 19:2024.08.19.608713. Preprint. [Version 1] doi: 10.1101/2024.08.19.608713 (PMC11475871; doi:10.1101/2024.08.19.608713)

Supplemental Fig 1. Cycloheximide feeding causes defects in oogenesis

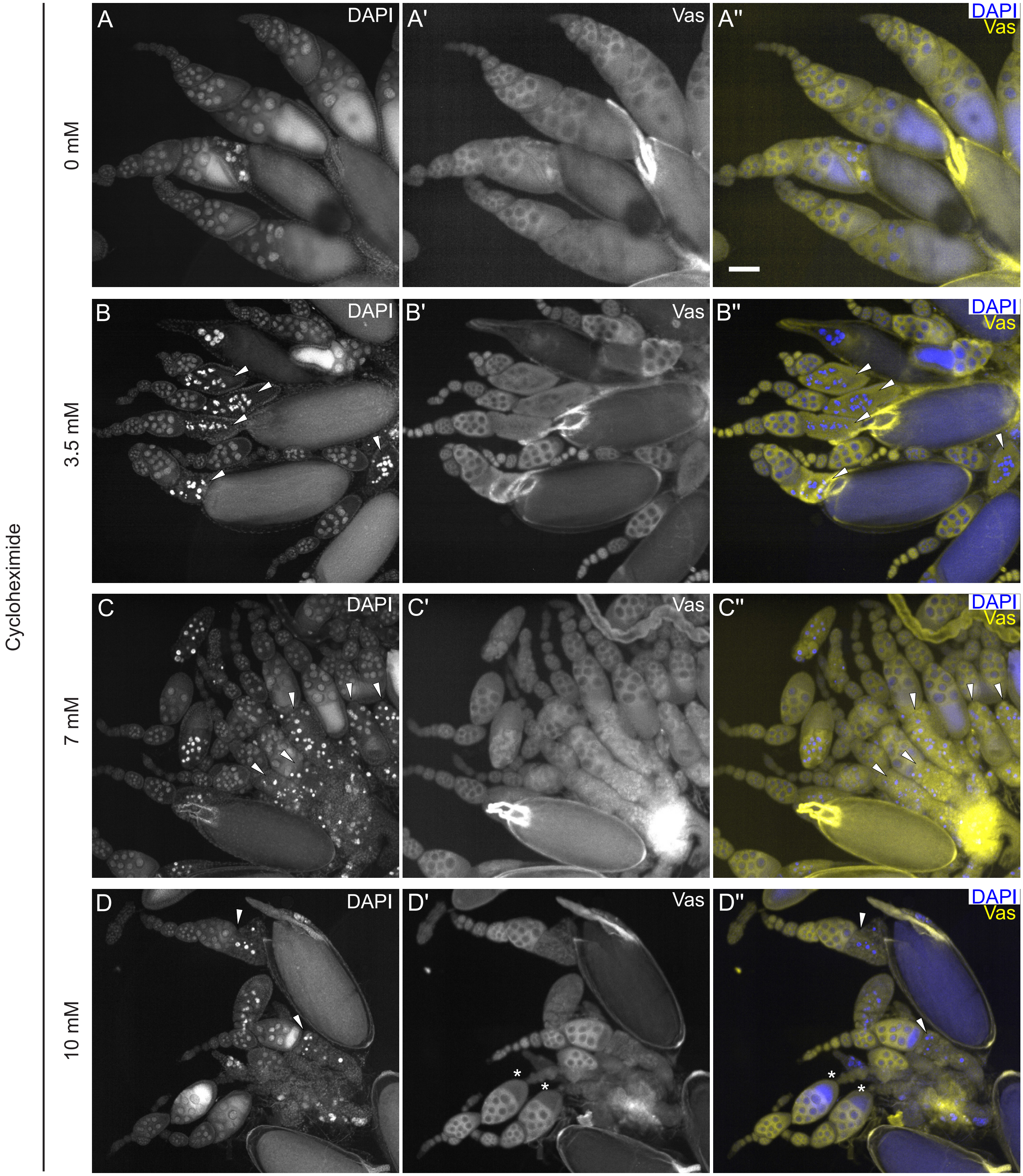

Supplemental Fig 2. Cycloheximide feeding affects ovary sizes and progeny development

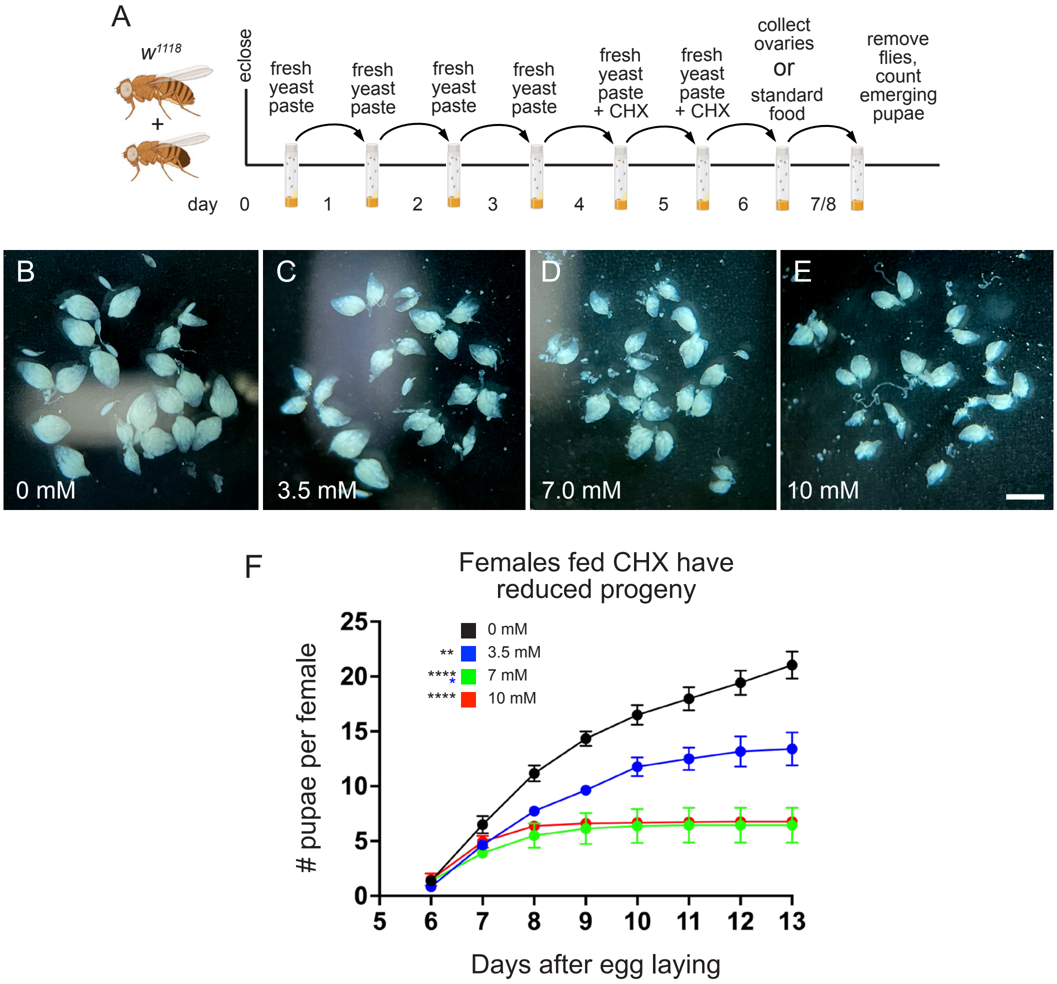

Supplement: Supplement 1 — Supplementary Fig 1. A rich diet supplemented with CHX causes defects in oogenesis. (A-D”) Dissected ovaries labeled with anti-Vasa to label germplasm and DAPI to label nuclei. Ovaries were dissected from females fed for 24 hrs with wet yeast paste containing (A-A”) 0 mM, (B-B”) 3.5 mM, (C-C”) 7.0 mM, and (D-D”) 10 mM CHX. White arrowheads indicate apoptotic cells (B-D”). Asterisks indicates ovarioles with missing follicles stages (D’, D”). (A, B, C, D) White = DAPI. (A’, B’, C’, D’) white = anti-Vasa. (A”, B”, C”, D”, merge) yellow = anti-Vasa, blue = DAPI. Scale bar = 100 μm in A” for A-D”. Supplementary Fig 2. A rich diet supplemented with CHX affects ovary size and progeny number. (A) Schematic illustrating the feeding regimen for comparison of ovary sizes in (B-E) and the pupation assay (F). Newly eclosed flies were fed freshly prepared yeast paste daily for four days, after which they were fed with fresh yeast paste containing CHX for 48 hrs. After CHX feeding, flies were dissected to obtain ovaries or transferred to a standard food vial and allowed to lay eggs for 48 hrs. Subsequently, the adults were removed from the vials and emerging pupae counted. (B-E) Ten pairs of ovaries from females fed with varying concentrations of CHX for 48 hrs. Females fed CHX have visibly smaller ovaries. (F) The percent pupae from females fed with varying concentrations of CHX for 48 hrs normalized to wild type. Percent pupae emerging for the flies fed with CHX-containing yeast paste were significantly lower. (G) Total protein amounts from ovaries after CHX feeding for 24 hrs. The protein amount was normalized with the ovary area (Fig 5F). Each points on the graph represent one replicate, and bars on the graph represent the average of all three replicates. All experimental conditions had three replicates. Error bars = SEM. Data were analyzed for significant differences by ANOVA followed by Tukey’s post hoc test. Asterisks in graphs indicate significance on day 9 after egg layin [file media-1.pdf]
